# Characterization and Validation of a Lyophilized Loop-Mediated Isothermal Amplification Method for the Detection of *Esox lucius*

**DOI:** 10.1007/s12010-023-04799-x

**Published:** 2023-12-28

**Authors:** Nivedhitha Jothinarayanan, Frank Karlsen, Lars Eric Roseng

**Affiliations:** Department of Microsystems, University of South-Eastern Norway, Raveien 215, 3184 Borre, Norway

**Keywords:** Loop-mediated isothermal amplification, Lyophilization, Freeze-dried beads, *Esox lucius*, Storage, Characterization and validation

## Abstract

In many ways, globalization is beneficial, but in one way, it promotes the spread of alien (invasive) species through international trade and transport. In different habitats, *Esox lucius* (northern pike) can be considered a regionally alien species, and this fish tends to establish a higher density population than desired in fresh water. Early identification of such invasive species using sensitive and quick methods is important to be able to take immediate measures and avoid environmental problems. Loop-mediated isothermal amplification (LAMP) has emerged as the best DNA/RNA detection technique, without any expensive equipment and could be used to detect environmental DNA (eDNA). However, the reagents for amplification are not stable at ambient temperature for field applications. Therefore, this work aims to lyophilize the entire reaction mixture as a single microbead, with enzyme, and LAMP primers towards the detection of mitochondrial cytochrome B (Cyt B), a housekeeping gene in *Esox lucius*. Analytical and molecular techniques were performed to characterize and validate the lyophilized beads, respectively. The lyophilized beads were stored at two different temperatures, at 20 °C and 4 °C, and tested for biological activity after different time intervals. The result shows that lyophilized beads are bioactive for almost 30 days when stored at 20 °C, while beads at 4 °C did not lose their bioactivity after storage for up to one year. This study will be particularly useful for conducting on-site LAMP analyses in the field, where resources for freezing and storage are limited.

**Graphical Abstract:**

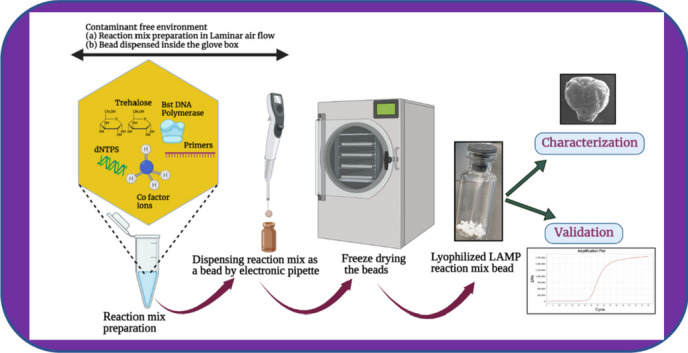

## Introduction

Invasion by unwanted species will have unexpected and undesirable consequences for ecosystems. The presence of alien species can be monitored and assessed at an early stage using an environmental DNA (eDNA) detection technique, rather than just being assessed as an exotic condition [1, 2]. eDNA is a genetic material that is released from species living in the habitat, into the environment including water, soil, and sediments. Protocols using eDNA can allow for rapid, cost-effective, and standardized collection of data on species distribution and relative abundance [3]. The quantitative evaluation of native species living in a habitat can be supported by the collection of background information on the invasive species, such as introduction routes into the habitat and changes in ecosystems. This provides better information about the total amount of native species and helps to execute essential procedures [4]. Globally, there is an increasing potential impact with high risk for the future because the biological invasions occur through routes of expanded trade and transport [5, 6]. Regarding the assessment of endangered species, the International Union for Conservation of Nature (IUCN) has expanded the global percentage of threatened species to 51% [7]. Consequently, prevention is more important than treatment. By identifying the new threats, this can help to guide future research. But it is impossible to perform accurate imaging surveys for invasive species in such water bodies; therefore, environmental DNA survey would be the best way to explore the presence of unwanted species [8]. Northern Pike (*Esox lucius*), which is a natural species in some water habitats in south-eastern parts of Norway, will also be a particularly undesirable species in Norwegian mountain lakes. The distribution of this species must therefore be carefully identified. An unwanted spread will largely destroy the local indigenous species such as trout and pearl mussel [9].

Detection of eDNA is an effective and feasible technique to monitor biodiversity and to assess the presence of different living organisms [10, 11]. In the absence of good tools to find the target species directly in the aquatic environment, eDNA monitoring is a suitable method for detecting DNA from species such as fish. The application of eDNA monitoring is used both in research and in the authorities’ management of aquatic species in freshwater, fjords, and marine ecosystems. Molecular biomarkers are required in eDNA analyses to ensure specific detection of the relevant target organism. The molecular biology techniques such as Polymerase chain reaction (PCR) [12], Loop-mediated isothermal amplification (LAMP) [13], Nucleic acid sequence-based amplification (NASBA) [14], and several other methods can be used in an eDNA evaluation. In this study, we have used the LAMP technique to analyze the DNA of the target species with a desired gene expression, because LAMP is an isothermal amplification method and more advantageous than PCR, when the process is to be performed in an automatic way on a lab-on-a-chip platform, in the field. The key feature of the LAMP technique is that, it amplifies nucleic acids under isothermal conditions in the range of 61–65 °C. LAMP is a simple and cost-effective reaction method. Another notable characteristic of the technique is good specificity and high amplification efficiency. Loss of activity is prevented, as there is no thermal change during the reaction, and this is the main reason for higher amplification efficiency. Additionally, in the aspect of greater specificity, the LAMP method uses four primers to identify the six different segments on the template DNA [13, 15, 16]. In this study, highly specific LAMP primers targeting the mitochondrial Cyt B gene of Northern Pike have been designed and used.

Although LAMP is a potential technique for DNA detection and is a cost-effective reaction, the reaction mixture consists of buffer ions, enzyme, deoxynucleotide triphosphates (dNTPs), betaine, primers of different concentration, and fluorescent dye, and this reaction mixture is considered not stable at room temperature. Transport of the LAMP reagents from one place to another in the field involves the maintenance of dry ice as well as transport costs. A major disadvantage will be the maintenance of the cold chain during the entire transport and storage [17]. Our study therefore focuses on constructing all necessary reaction components in a freeze-dried bead form, which is well suited for transport and storage. Such freeze-dried reagent beads can be easily placed inside a reaction chamber of lab-on-a-chip platform.

Lyophilization is the best process for preserving biomolecules such as proteins, enzymes, and pharmaceutical liposomes, including a disaccharide as a key protective agent. The freeze-drying process consists of two main steps, namely freezing and drying under vacuum. The drying process itself is further classified into (i) the primary drying where frozen water is removed and (ii) the secondary drying where unfrozen (bound) water is removed [18]. At the time of drying, when water molecules evaporate, the tertiary biostructure of the protein will be destroyed, and this is avoided by the adding a sugar as a non-specific protein stabilizer. Commonly, named disaccharides such as sucrose and trehalose are used as cryoprotectants [19, 20]. In this study, we used trehalose which is a good glass former that helps to stabilize the biomolecules during and after the freeze-drying. The important glass transition factor of disaccharides is high in trehalose and acts as a support under higher temperature storage condition. Other additional properties should also be advantageous such as (i) absence of internal hydrogen bond, (ii) less hygroscopicity, and (iii) low chemical reactivity. This has been shown in previous studies by experimenting the stability of DNA modifying enzyme with 0.3 M trehalose compared to other saccharides such as sucrose, sorbitol, mannitol, and galactitol [21]. Studies have revealed that DNA damage will be significantly reduced during the freeze-drying process of biomolecules, although DNA damage could not be avoided during room temperature storage. The damage can still be reduced in the presence of trehalose. The cause of DNA destruction is due to free radical-mediated oxidation. Stability is maintained based on the residual moisture content of the sample or on the presence and concentration of a cryoprotectant such as trehalose [22, 23].

The aim of the study is to determine the bioactivity of lyophilized LAMP reagent beads after storage for varying periods of time at two different temperatures. Batches of lyophilized LAMP reagents with designed primers and different trehalose concentrations were prepared, characterized, and stored at 4 °C and 20 °C. The result will provide the necessary information to develop freeze-dried reagents that can be placed, stored, and used inside a lab-on-a-chip platform, for the detection of eDNA from different species, in automated machines in the field. The research paper reveals the properties and stability of lyophilized LAMP reagents for the detection of *Esox lucius* DNA.

## Materials and Methods

### Lamp Reaction Setup for Lyophilization

The warm start LAMP Kit (New England Biolabs, Massachusetts) was employed for the reaction setup, and the components in the kit were Warm Start LAMP 2X Master mix and LAMP fluorescent dye. The master mixture is expected to contain 10 mM dNTPs mix, 8 U *Bst* DNA polymerase, 20 mM Tris HCl, 50 mM KCl, 0.1 % Tween, 10 mM (NH_4_)_2_SO_4_, 0.8 M betaine, and 8 mM MgSO_4_, and fluorescent dye could be FAM/SYBr Green for one reaction of amplification. Different concentration of trehalose includes 10 %, 15 %, and 20 % which was incorporated in the LAMP reaction mixture (w/v), containing optimized concentration of primers, enzyme, and cofactor ions that were subjected to the freeze-drying process. The reaction was carried out in 25 μL volume, as suggested by the warm start manual. All the mixture was prepared inside the laminar air flow chamber (ESCO Biosafety Cabinet, Nordic safe), to avoid contamination. The primers used in the reaction are constructed by LAMP designer (PREMIER Biosoft, USA) and are as follows: TACACCACAGGGCTTGATA; GCATGGGCTGTAACGATAA; AGGGTGCCAATATCTTTGTGGTTCTCAGCCATCCTACCTG; AGTCGGCACAGCCTTAAGCCTGGTCGTCACCTAAGAGA; ATCAGCGTGTGATTGCCA; and CCGAACTAAGCCAGCCAG at F3, B3, FIP, BIP, Loop F, and Loop B primer region, respectively [24], and commercially purchased from Eurofins Genomics (Denmark).

### Freeze Drying Procedure

The whole process of preparing frozen beads was carried out inside the glove box (MBraun EasyLab) to maintain the inert state with the aim of limiting contamination. The prepared LAMP reaction mixture was taken in an electronic pipette (eVol **XR**) capable of dispensing 5 μL volume bead in each turn. The beads were dispensed in cold-conditioned vials, placed in a metal block with a supply of liquid nitrogen. Then, frozen beads containing vials were transferred into the freeze dryer (Labconco, FreeZone Triad) with preprogrammed segments. The freeze-drying program with different segments and time duration is shown in Table 1.

**Table 1:** The freeze-drying program segments followed for the LAMP reaction mixture during drying

Segment	Ramp (°C/min)	Hold (°C)	Time (hr)	Vacuum (mbar)
Pre-freeze			6	~0.002 to 0.003
1	1	−55	3	
2	0.5	−10	2	
3	1	10	1	
4	1	20	1	
5	1	25	4	

### Analytical Characterization of Lyophilized Beads

The morphology of the lyophilized beads was observed under scanning electron microscope (SEM) (SEM Hitachi, SU 3500). The accelerated voltage of around 10 to 15 kV was used for observation at different magnification. To distinguish between the functional groups, Raman spectroscopy (Thermo Fisher, Nicolet iS50) was used for pure trehalose bead and the bead with LAMP reagent and trehalose at different concentration. The He-Ne laser is used for the Raman spectra measurement.

### LAMP Lyophilized Bead Validation

The prepared beads were stored at two different temperature conditions, one at room temperature 20 °C and another at 4 °C in a refrigerator. Then, five beads were rehydrated with 24 μL of RNase-free water and tested towards *Esox lucius* DNA template (1 μL) at different time periods; and the amplification cycle was performed using Applied Biosystems, StepOnePlus (Thermo Fisher Scientific, USA). The reaction was carried out at 65 °C for 1 h and set with 80 cycles. The amplicons were also quantified by fluorometer (Invitrogen, Qubit 4 Fluorometer) and viewed in 1 % agarose gel electrophoresis with ladder.

### Statistical Analysis

All the data were represented as ± standard error of the mean from *n* = 5 experiments. The statistical significance was considered using a sample *t*-test among each experiment from the Minitab 21 software. The probability value (*p*-value) 0.05 was considered statistically significant.

## Results

**Fig. 1:**
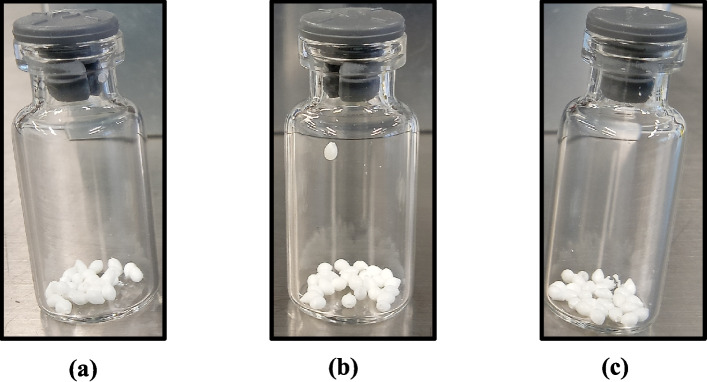
The LAMP mixture beads in different concentrations of trehalose (**a**) 10 %, (**b**) 15 %, and (**c**) 20 % weight percent

In this study, the process of making bioactive freeze-dried LAMP beads was developed to establish knowledge about long-term storage with the desired result. The lyophilized beads will be used as reagents inside a lab-on-a-chip platform in an automated analysis system. The purpose is to carry out on-site environmental monitoring of rivers and lakes using LAMP, and to detect the eDNA from *Esox lucius* (regionally alien). The specificity and sensitivity of the designed *Esox lucius* LAMP primers, which were used in the lyophilized beads, have been previously determined [24]. Here, some of the characteristic properties of the freeze-dried beads were examined. Three trehalose concentrations, 10 %, 15 %, and 20 % (w/v), were used in the LAMP mixture beads (Fig. 1). Several batches of 5 μL lyophilized beads were stored over time, in separate sealed glass ampoules both at room temperature 20 °C and at 4 °C in a refrigerator.

### Surface Dimensions and Morphology

**Fig. 2:**
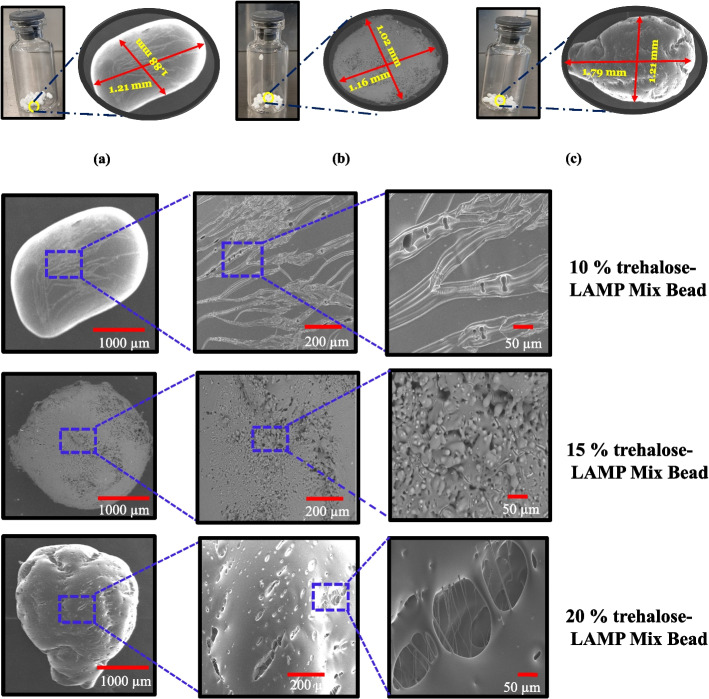
(**i**) The dimensions of the 5 μL lyophilized beads (a) 10 % trehalose- LAMP mixture bead, (b) 15 % trehalose-LAMP mixture beads, and (c) 20 % trehalose-LAMP mixture beads. The dimensions vary slightly for the beads. (**ii**) The surface morphology of the trehalose-LAMP mixture beads from lower to higher magnification

A scanning electron microscope was used to observe the objects in the micron to nano level range. The prepared beads were subjected to SEM observation and their dimension was marked and surface morphology observed (Fig. 2 (i) and (ii)). The physical shape of the 5 μL beads was found to be round to oval shape, depending on the needle tip and dispensing time. The dimension of the lyophilized beads was found to be in the range of 1.1 to 1.8 mm among the three trehalose concentration beads (Fig. 2 (i)). The surface nature of the lyophilized LAMP mixture beads was examined at higher magnification, revealing the dense and porous nature, various nanofiber formations connecting the voids of the beads, as clearly seen (Fig. 2 (ii)). It was noticed that a trehalose concentration of 10 % in the LAMP mixture beads readily absorbs moisture and shrinks immediately, while the increasing concentration of 15 % and 20 % was more stable when exposed to atmospheric air. In this context, the shrinkage pattern of the surface is seen for the 10 % trehalose-LAMP mixture bead, and a dense structural surface with a porous nature is visible for both the 15 % trehalose-LAMP mixture bead and 20 % trehalose-LAMP mixture bead. The freeze-dried LAMP beads, regardless of the trehalose concentration, appeared similar, yet the surface morphology varied between the different beads, and this was the case both with the same and with different trehalose concentrations. It was of interest to investigate the possible difference that could be linked to the morphology of the freeze-dried beads and to the formation of varieties of networks through glycosidic linkage, based on the difference in concentrations of the trehalose sugar. It was found that the different structure of the freeze-dried LAMP beads did not have a significant contribution to the reaction time of the LAMP mixture.

### Molecular Vibrations by RAMAN Spectroscopy

**Fig. 3:**
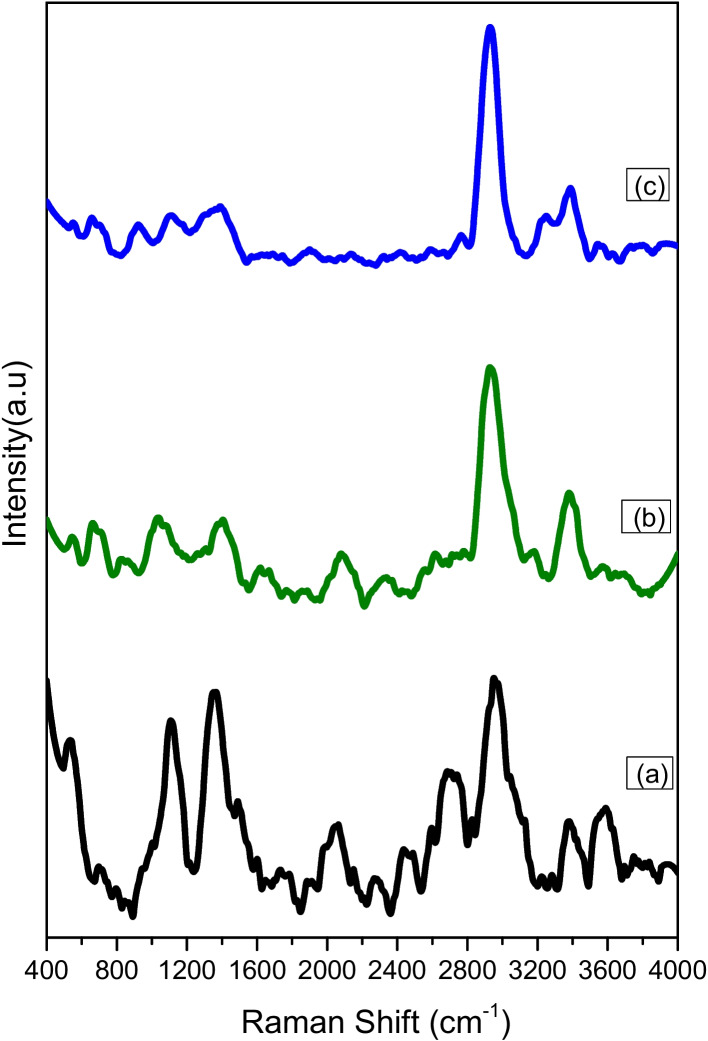
The Raman spectra for (**a**) pure trehalose bead, (**b**) the 15 % trehalose-LAMP mixture bead, and (**c**) the 20 % trehalose-LAMP mixture bead

Trehalose-water-protein interactions have been previously and extensively studied using spectroscopic methods. In particular, Raman spectroscopy has been used because of the advantage of label-free detection in biological samples [25–27]. RAMAN spectra analysis was therefore performed on different freeze-dried beads; completely pure trehalose beads, and some of the trehalose-LAMP mixture beads, and the spectra are given (Fig. 3). In the completely pure trehalose bead, the vibrations in the C-O-C skeletal structure produced strong C-C bond stretching peaks in the range of 400 to 1800 cm^−1^, which is considered to be a fingerprint region of trehalose. Clearly prominent peaks at 529 cm^−1^, 1105 cm^−1^, and 1363 cm^−1^ (Fig. 3 (a)) are also characteristic of trehalose. Addition of enzyme and organic matter resulted in a change of the intensity of trehalose in the fingerprint region (Fig. 3 (b) and (c)). The intensity of the shoulder peak in the region around 2900 cm^−1^, which corresponds to C-H stretching, is different for completely pure trehalose beads and for trehalose-LAMP mixture beads, and this also reinforces the presence of biomolecules. The region of ~3400 cm^−1^ indicates O-H symmetric and anti-symmetric stretching because free water may be present during measurement [28–30]. From these RAMAN spectra, the difference in the molecular vibration of both completely pure trehalose beads and trehalose-LAMP mixture beads can be seen. In addition, the literature on Raman spectroscopy describes that trehalose has an ability to make the protein inflexible, which is an essential factor for protein stabilization [31]. This has also been observed in our results of trehalose both with and without biomolecules.

### Stability Study of Lyophilized Bead

**Fig. 4:**
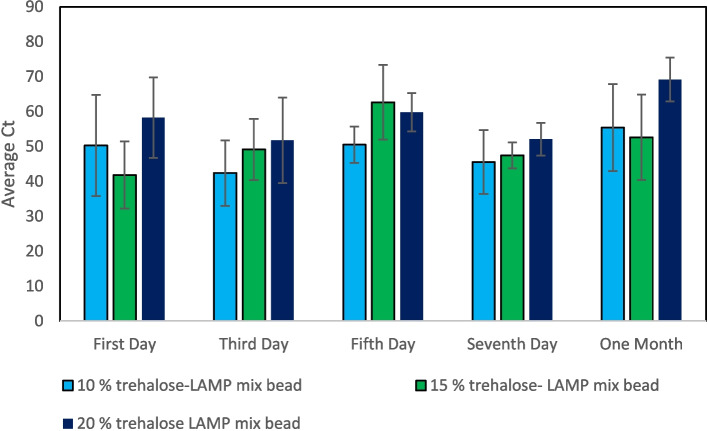
Exhibits the Ct values obtained from the 20 °C stored lyophilized LAMP beads at different time intervals

The trehalose-LAMP mixture beads were stored at room temperature to evaluate the activity of lyophilized reagents at different time intervals and the average Ct values (cycle threshold) of the amplification is indicated (Fig. 4). Our results reveal that the duration of the stability of the lyophilized reagents without losing any sensitivity was up to 30 days at room temperature storage. The bioactivity was completely lost after 30 days and showed zero Ct for 2 months of storage. The average Ct value for 10 % trehalose-LAMP mixture bead is 50.3, 42.4, 50.5, 45.5, and 55.4 for storage after the first day, third day, fifth day, seventh day and one month, respectively. After storage of one month, the average Ct for 15 % trehalose-LAMP mixture bead is slightly less than 10 % trehalose-LAMP mixture beads and much less than 20 % trehalose-LAMP mixture beads. The 95% confidence interval for the population mean of the 15 % trehalose-LAMP mixture beads was considerably less in one month storage, compared to the other two compositions of beads as shown in Table 2 (a), (b), and (c). Only the 15 % trehalose-LAMP mixture beads had a significantly lower Ct, when stored at room temperature for one month. Although the 10 % trehalose-LAMP mixture beads showed effective bioactivity over the entire storage period, these beads are not physically stable at room temperature because they readily absorb moisture and become hygroscopic. Besides, 15 % and 20 % trehalose-LAMP mixture beads maintain their physical bead shape to a greater extent, even when exposed to atmospheric moisture.

**Table 2:** Shows descriptive statistics and sample *t*-test for trehalose-LAMP mixture beads, stored at room temperature

Sample	*N*	Mean	StDev	SE mean	95% CI for μ	Null hypothesis	H_0_: μ = 0.05
						Alternative hypothesis	H_1_: μ ≠ 0.05
						*T*-value	*P*-value
(a) 10 % trehalose-LAMP mixture beads							
First day	5	50.30	14.46	6.47	(32.35; 68.26)	7.77	0.001
Third day	5	42.40	9.38	4.19	(30.75; 54.04)	10.10	0.001
Fifth day	5	50.54	5.18	2.32	(44.11; 56.98)	21.78	0.000
Seventh day	5	45.54	9.13	4.08	(34.21; 56.88)	11.14	0.000
One month	5	55.43	12.46	5.57	(39.97; 70.90)	9.94	0.001
(b) 15 % trehalose-LAMP mixture beads							
First day	5	41.83	9.63	4.31	(29.87; 53.79)	9.70	0.001
Third day	5	49.18	8.79	3.93	(38.26; 60.09)	12.49	0.000
Fifth day	5	62.65	10.68	4.77	(49.39; 75.90)	13.11	0.000
Seventh day	5	47.76	3.74	1.67	(43.11; 52.41)	28.50	0.000
One month	5	52.63	12.19	5.45	(37.49; 67.77)	9.64	0.001
(c) 20 % trehalose-LAMP mixture beads							
First day	5	58.30	11.52	5.15	(44.00; 72.60)	11.31	0.000
Third day	5	51.74	12.26	5.48	(36.52; 66.97)	9.43	0.001
Fifth day	5	59.81	5.49	2.46	(52.98; 66.63)	24.32	0.000
Seventh day	5	52.10	4.67	2.09	(46.30; 57.89)	24.94	0.000
One month	5	69.17	6.26	2.80	(61.39; 76.95)	24.67	0.000

μ: population mean of first day; third day; fifth day; seventh day; one month

**Fig. 5:**
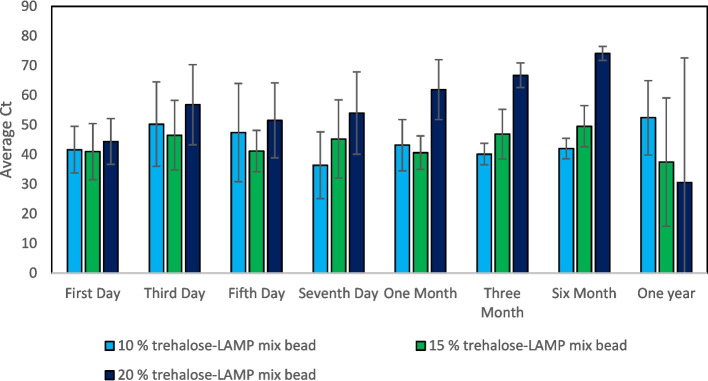
The Ct values obtained from the 4 °C stored lyophilized LAMP beads at different time intervals

The trehalose-LAMP mixture beads were also refrigerated (4 °C) over time to evaluate the bioactivity of lyophilized reagents at different time intervals, and the average Ct values of the amplification were calculated (Fig. 5). We observed that the activity of lyophilized beads was not lost after one year storage at 4 °C and the statistical significance is shown in Table 3 (a), (b), and (c) for 10 %, 15 %, and 20 % trehalose-LAMP mixture bead, respectively. Although the 20 % trehalose-LAMP mixture bead has a lower Ct value, our results showed that these values are not consistent and had a standard error of ± 42. The bioactivity of the 15 % trehalose-LAMP mixture beads was significant after one year of storage with a lower Ct value.

**Table 3:** Shows descriptive statistics and sample *t*-test for trehalose-LAMP mixture beads, stored at 4 °C

Sample	*N*	Mean	StDev	SE mean	95% CI for μ	Null hypothesis	H_0_: μ = 0.05
						Alternative hypothesis	H_1_: μ ≠ 0.05
						*T*-value	*P*-value
(a) 10 % trehalose-LAMP mixture beads							
First day	5	41.66	7.91	3.54	(31.84; 51.49)	11.76	0.000
Third day	5	50.28	14.23	6.36	(32.62; 67.95)	7.90	0.001
Fifth day	5	47.41	16.55	7.40	(26.86; 67.96)	6.40	0.003
Seventh day	5	36.42	11.25	5.03	(22.45; 50.40)	7.23	0.002
One month	5	43.16	8.65	3.87	(32.43; 53.90)	11.15	0.000
Three months	5	40.17	3.66	1.64	(35.62; 44.72)	24.50	0.000
Six months	5	42.05	3.49	1.56	(37.71; 46.39)	26.87	0.000
One year	5	52.41	12.56	5.62	(36.81; 68.01)	9.32	0.001
(b) 15 % trehalose-LAMP mixture beads							
First day	5	41.00	9.47	4.24	(29.24; 52.77)	9.66	0.001
Third day	5	46.53	11.75	5.25	(31.95; 61.12)	8.85	0.001
Fifth day	5	41.18	6.95	3.11	(32.56; 49.81)	13.24	0.000
Seventh day	5	45.25	13.17	5.89	(28.89; 61.60)	7.67	0.002
One month	5	40.65	5.61	2.51	(33.68; 47.61)	16.18	0.000
Three months	5	46.92	8.38	3.75	(36.51; 57.33)	12.50	0.000
Six months	5	49.57	6.96	3.11	(40.92; 58.21)	15.90	0.000
One year	5	37.45	21.65	9.68	(10.57; 64.33)	3.86	0.018
(c) 20 % trehalose-LAMP mixture beads							
First day	5	44.42	7.71	3.45	(34.85; 53.99)	12.87	0.000
Third day	5	56.82	13.51	6.04	(40.04; 73.60)	9.39	0.001
Fifth day	5	51.50	12.66	5.66	(35.78; 67.21)	9.09	0.001
Seventh day	5	54.01	13.85	6.19	(36.81; 71.21)	8.71	0.001
One month	5	61.95	10.11	4.52	(49.40; 74.51)	13.69	0.000
Three months	5	66.74	4.13	1.85	(61.60; 71.87)	36.08	0.000
Six months	5	74.12	2.35	1.05	(71.20; 77.04)	70.42	0.000
One year	5	30.6	42.0	18.8	(−21.6; 82.8)	1.62	0.180

μ: population mean of first day; third day; fifth day; seventh day; one month; three months; six months; one year

### Quantification and Gel Electrophoresis

**Fig. 6:**
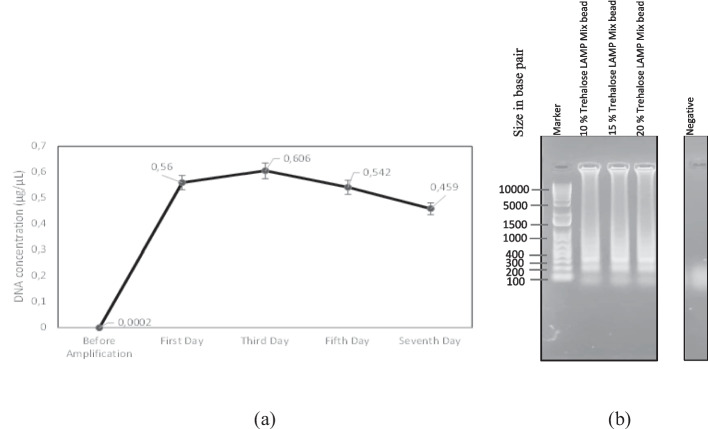
The estimation of DNA concentration (**a**) quantification of DNA before and after amplification with 15 % trehalose-LAMP reagent bead mixture stored at room temperature, and (**b**) appearance of smear DNA band after amplification with lyophilized beads and compared with the negative control and 1 Kb DNA ladder (Marker)

The template of Northern Pike (*Esox lucius*) DNA was quantified both before and after the amplification reaction and amplicons were seen in 1 % agarose gel (Fig. 6 (a) and (b)). Before amplification, the Northern Pike DNA concentration was 0.0002 μg/μL and this concentration increases drastically after amplification with lyophilized LAMP reagent beads. The results indicate that the number of DNA copies would be slightly reduced on day seven of storage of LAMP beads at room temperature. The LAMP amplicons were also observed as smear band in agarose gel electrophoresis together with the 1Kb ladder and negative control.

## Discussion

Early detection of *Esox lucius* (a regionally alien species) in local habitats is particularly important to provide guidance and to assess the implementation of measures early as a part of environmental monitoring. The lyophilized LAMP beads described in this study could have a significant impact on establishing a better system for real-time on-site environmental monitoring in the field. The development of lyophilized LAMP assay for the detection of *Esox lucius* using a lab-on-a-chip platform in combination with an automatically operated electronic instrument will enable efficient and rapid environmental monitoring.

LAMP has the properties that make this molecular biological method well suited for fieldwork studies, and LAMP is likely to be chosen for automatic real-time on-site environmental monitoring. Unlike PCR, where different temperature patterns are required for DNA amplification to occur, only a constant temperature around 60–65 °C is required for LAMP. This means that the LAMP process is much better suited than PCR, when a process for automatic DNA amplification is to be established inside a lab-on-a-chip platform. The design of the chip, and the automatic control of the amplification process inside the chip will be easier to establish when only a constant temperature is obliged.

Our results show that lyophilized LAMP reagent beads, containing 15 % by weight of the disaccharide trehalose, will maintain their bioactivity after storage at 20 °C for one month and for up to a year after storage at 4 °C. The amount of target DNA is inversely related to the Ct value and a lower Ct value with efficient amplification implies the required concentration of template. On the other hand, very excessive concentration of template DNA affects the efficiency of amplification and results in large range of Ct variation. However, assay-independent factors affecting the Ct values are less in this experiment. The 10 % trehalose-LAMP mixture bead which readily absorbs atmospheric moisture might fail to protect the entire enzyme assembly and primer sequence while in storage and is reflected in the insignificance of Ct value. At the same time, higher concentration of 20 % trehalose will form a macromolecular cluster that can interact with itself and be described as a crowding agent [32]. This can reduce the amplification rate and affects the Ct value. Therefore, the result in this study recommends the optimized concentration of 15 % trehalose in the LAMP mixture beads, when Northern Pike is to be detected with a significant Ct value.

Previous studies of lyophilized LAMP reagents to detect dengue virus [33] and Leptospira [34] have also reported the stability after different storage conditions. These studies reported that lyophilized reagents will break the cold chain associated with storage and that the LAMP reagents bioactivity will be effective over time. The importance of lyophilized reagents inside lab-on-a-chip consumables will help to overcome issues such as (i) simplifying reagent storage requirements and (ii) minimizing user intervention, thereby paving the way for sensitive molecular technologies for non-experts. LAMP has characteristics that make it ideal for field work and is likely to be used in real-time on-site environmental monitoring.

Earlier reports on the stabilization and modulation of enzymes are based on the ability of the disaccharide to form hydrogen bonds, and this type of interaction will preferentially stabilize the enzyme. The size exclusion effect is higher for trehalose, which contributes to strong stabilization properties [35]. The results of our study indicate that the stabilization property is directly proportional to the concentration of trehalose, and it is beneficial when the lyophilized beads are to be stored under normal conditions after the freeze-drying process. We observed that higher concentration of trehalose profoundly influences the efficiency of nucleic acid amplification. Trehalose is an effective disaccharide as a stabilizer, but it is also an inhibitor of several enzymes as mentioned in the literature [35–37]. When a 20 % by weight of trehalose was used in our LAMP mixture beads, the results also suggest it can lower efficiency.

The advantage of the proposed lyophilized reagent method in this article is (1) to secure bioactive LAMP mixture reagents after storage at relatively high temperatures over time and (2) to reduce the likelihood of contamination by avoiding the need to open the reaction tube when assays are performed on site in field situation. Our results show that a trehalose concentration of 15 % by weight in the lyophilized LAMP mixture beads will be the best trehalose concentration. Compared to the 10 % and 20 % trehalose beads, the 15 % trehalose-LAMP mixture beads will be well suited for both room temperature storage and efficient bioactivity. Our freeze-dried beads were specially designed with target-specific LAMP primers for the detection of mitochondrial Cyt B gene in *Esox lucius*. The use of such lyophilized beads avoids the errors that can occur when such reaction mixtures are prepared manually in the field as part of the environmental DNA analysis. In addition, such lyophilized beads can be used as the LAMP mixture reagents placed inside automated lab-on-a-chip platforms, without manual handling and can easily replace the PCR-based field studies, where resources are limited, and when the field samples must be transported back to the laboratory for analysis.

## Conclusions

The results indicate that lyophilized LAMP reaction mixture beads will be bioactive for almost 30 days, after storage at room temperature, and that the bioactivity of freeze-dried beads will last longer when stored at lower temperatures. When stored in a refrigerator at 4 °C, the freeze-dried beads were bioactive for one year. This indicates that lyophilized state LAMP reagents can be placed on a lab-on-a-chip platform and that it is possible to store ready packaged chips with reagents over time. This is obviously useful information for the development of automatic systems that independently (without human intervention) perform LAMP analyses in the field for detection of northern pike. The lyophilized LAMP beads easily dissolve in RNase-free water and can be readily mixed with the desired DNA template for LAMP analyses. Being able to eliminate the maintenance of cold storage conditions during shipping and storage also provides flexibility for field workers to perform LAMP analyses under field conditions.

## Data Availability

Not applicable.
